# Attentional Bias Modification With Serious Game Elements: Evaluating the Shots Game

**DOI:** 10.2196/games.6464

**Published:** 2016-12-06

**Authors:** Wouter J Boendermaker, Soraya Sanchez Maceiras, Marilisa Boffo, Reinout W Wiers

**Affiliations:** ^1^Utrecht UniversityDepartment of Child and Adolescent StudiesUtrechtNetherlands; ^2^Department of Developmental PsychologyUniversity of AmsterdamAmsterdamNetherlands

**Keywords:** cognitive training, cognitive bias modification (CBM), attentional bias, adolescents, serious games, motivation

## Abstract

**Background:**

Young adults often experiment with heavy use of alcohol, which poses severe health risks and increases the chance of developing addiction problems. In clinical patients, cognitive retraining of automatic appetitive processes, such as selective attention toward alcohol (known as “cognitive bias modification of attention,” or CBM-A), has been shown to be a promising add-on to treatment, helping to prevent relapse.

**Objective:**

To prevent escalation of regular use into problematic use in youth, motivation appears to play a pivotal role. As CBM-A is often viewed as long and boring, this paper presents this training with the addition of serious game elements as a novel approach aimed at enhancing motivation to train.

**Methods:**

A total of 96 heavy drinking undergraduate students carried out a regular CBM-A training, a gamified version (called “Shots”), or a placebo training version over 4 training sessions. Measures of motivation to change their behavior, motivation to train, drinking behavior, and attentional bias for alcohol were included before and after training.

**Results:**

Alcohol attentional bias was reduced after training only in the regular training condition. Self-reported drinking behavior was not affected, but motivation to train decreased in all conditions, suggesting that the motivational features of the Shots game were not enough to fully counteract the tiresome nature of the training. Moreover, some of the motivational aspects decreased slightly more in the game condition, which may indicate potential detrimental effects of disappointing gamification.

**Conclusions:**

Gamification is not without its risks. When the motivational value of a training task with serious game elements is less than expected by the adolescent, effects detrimental to their motivation may occur. We therefore advise caution when using gamification, as well as underscore the importance of careful scientific evaluation.

## Introduction

Heavy alcohol use during adolescence and early adulthood has been related to health problems and academic underperformance [1] and is an important predictor of addictive behaviors later in life [2]. Dual-process models of addiction [3,4] suggest that prolonged use of alcohol, especially when initiated during adolescence [5], can lead to the development of strong automatically triggered reactions toward alcohol, which in turn facilitate the development of addictive behaviors. This is visible in heavy alcohol users’ tendency to approach [6] and selectively attend to [7] alcohol-related stimuli more quickly, compared with non-alcohol-related stimuli. Opposite to these strengthened automatic reactions are reflective cognitive processes, including control abilities (eg, working memory [8], inhibition [9]) that can be too weak or too late to moderate the automatically triggered reactions [5]. The resulting imbalance between automatically triggered appetitive processes and reflective control processes may contribute to escalation in problem drinking.

Research has shown that, in both longtime heavy users and clinical patients, both types of processes can successfully be trained or retrained, resulting in less craving and lower relapse rates [7,10-13]. Despite these promising results, application of cognitive training in younger populations has proven more difficult [14], for a number of reasons. First, youngsters tend to perceive stronger positive than negative effects of their alcohol use [15], perhaps because positive effects of alcohol tend to occur sooner than the negative effects [16], making those positive associations stronger. This typically results in lower motivation to change their (drinking) behavior compared with patient populations. Second, the fact that most training paradigms are long and often viewed as tedious and boring [17] adds to the problem. To improve motivation to train, one potential solution could be to make the training sessions more fun to do by adding game elements into the training paradigm. For example, Dovis and colleagues [18] offered children with attention-deficit/hyperactivity disorder (ADHD) a computer game version of a working memory task and observed that they normalized their persistence of performance to the level of children without ADHD. Dennis and O’Toole [19] used a mobile game based on attentional bias modification training as an intervention for stress and anxiety in highly trait-anxious university students and showed a significant reduction in threat bias.

In this paper, we apply similar gamification techniques to a typical cognitive bias modification of attention (CBM-A) training task aimed at training attention away from pictures of alcoholic beverages: the visual probe task (VPT) [20,21]. In the VPT participants are shown pairs of pictures, one of a relevant stimulus (eg, a picture of an alcoholic beverage), the other a visually similar, neutral stimulus (eg, a picture of a nonalcoholic beverage). Next, a probe (eg, an arrow pointing up or down) appears at the location of one of the stimuli, and the instruction is to quickly identify the probe (eg, respond to the direction of the arrow). The contingency between the location of the probe and the stimulus it replaces can be manipulated. To assess attentional bias, the probe appears equally often at the location of both stimulus types; to train attention away from a certain set of stimuli (eg, alcohol), the probe appears more often at the location of the other set of stimuli (eg, nonalcoholic). Schoenmakers and colleagues [21] showed that CBM-A can indeed increase the ability to disengage from alcohol-related cues and found that alcohol-dependent patients who had received the CBM-A training took significantly longer to relapse after training than patients who had not received CBM-A.

It should be noted that although adding game elements to a cognitive training task may help increase participants’ motivation to train, they usually also influence the specific task features and parameters and inevitably change to some degree the evidence-based nature of the task [22]. For example, using points as rewards in the gamified training in order to enhance motivation to train may also counterbalance intrinsic motivation to train. As such, we will compare the new gamified VPT training (VPT-G) with both a regular VPT training (VPT-R), to evaluate the added motivational effects of the game elements, as well as with a placebo version of the regular VPT training (VPT-P) to establish whether it has a training effect similar to the VPT-R. This results in the following hypotheses. First, we expect a significant reduction in attentional bias toward alcohol stimuli in both the VPT-R and VPT-G conditions compared with the VPT-P condition (H1). This will be measured using both an assessment version of the VPT and another task that also measures attentional bias but is procedurally different, that is, the visual search task (VST) [23]. Next, we expect the same pattern of results between conditions with regard to decline in actual drinking behavior (H2). Finally, we expect to see that motivation to train is positively affected by the training in the VPT-G condition but not in the VPT-R and VPT-P conditions (H3), while motivation to change is expected to remain unaffected as it is not explicitly targeted by this training (H4).

## Methods

### Design and Procedure

The training consisted of 4 sessions, at least 1 day apart, over the course of 2 weeks. The first and last training sessions were combined with the assessment tasks in our laboratory; the 2 remaining training sessions were done at home. During the first session, participants were informed about the study’s goal, gave digital informed consent, and were randomly assigned to the VPT-P (n=33), VPT-R (n=30), or VPT-G (n=33) condition. They continued with digital versions of the Alcohol Use Disorders Identification Test (AUDIT), a short Readiness to Change Questionnaire (RCQ), the Timeline Followback (TLFB) questionnaire, the Alcohol Use Questionnaire (AUQ), and a Motivation to Train Questionnaire (MTQ). After the questionnaires, they completed the VPT and VST baseline assessment and finished the session with the first VPT training. The following second and third sessions solely consisted of the VPT training task. The last session started with the fourth VPT training, after which they performed the VPT and VST posttraining assessments and the AUQ, TLFB, RCQ, and MTQ questionnaires, supplemented by a brief set of questions about the training itself (EVAL). To evaluate drinking behavior after the training, a follow-up TLFB was filled in via email 2 weeks after session 4.

### Participants

A sample of undergraduate students (n=96, mean age 21.2 years, SD 1.8, range 18-28 years, 71%, 68/96 female) was recruited through the university laboratory’s website, based on their drinking behavior (≥5 standard glasses of alcohol on average per week for males; ≥4 for females). Participants received study credits or €30 for taking part in the experiment. The study was approved by the Ethics Committee of the University of Amsterdam (Protocol Number: 2015-DP-4215).

### Materials

*Alcohol use and problems* were measured with 3 questionnaires: A TLFB [24,25] was used to measure alcohol consumption per day over the past week and also included a question about the number of binge drinking occasions during the past 30 days (>5 standard glasses of alcohol consumed during one occasion for male participants; >4 for females). An adapted version of the AUQ [26] was used to assess drink-specific alcohol consumption over the past 6 months. For analyses, Mehrabian and Russell’s [26] equation 2 was used to calculate the habitual alcohol consumption (HAC), including those items regarding consumption of beer, wine, and liquor, as well as our added items concerning alcohol pops. Alcohol-related problems were measured with the AUDIT [27], which includes 10 multiple-choice questions regarding alcohol consumption and alcohol-related problems. The overall AUDIT score ranges between 0 and 40, with ≥8 indicating an increased risk of alcohol-related problems in normal samples and ≥11 in student samples [28].

*Motivation to train* was assessed using a self-developed 4-item questionnaire, each item rated on a 5-point Likert scale ranging from “strongly disagree” to “strongly agree.” *Motivation to change* was assessed using a shortened version of the RCQ [29], consisting of 3 multiple-choice items. The EVAL questions concerned how they rated the training overall. See Multimedia Appendix 1 for an overview of translated questions.

*Attentional bias* was measured using assessment versions of the VST and VPT paradigms. In the VST [23,30], participants were shown a grid of 4 by 4 pictures of beverages, where only 1 was of a different type: 1 alcoholic among 15 nonalcoholic beverages, or vice versa. The instruction was to find and click the deviant type of beverage as quickly and accurately as possible. To focus visual attention to the center of the grid, each trial started with a fixation cross in the center of the screen the participants had to mouse over in order to start the trial. When an incorrect response was given, feedback was given and the trial had to be redone. The task consisted of 6 blocks of 18 trials, using active (person drinking) or passive (bottle or glass only) pictures of alcoholic and nonalcoholic beverages or neutral pictures of 5- or 7-petaled flowers, following the schedule in Table 1. The order of the blocks containing beverages was counterbalanced over participants. A progress bar indicated the number of trials left in each block. The attentional bias scores for active and passive stimuli were computed by subtracting the respective average reaction times for selecting alcoholic beverages from the average reaction times for selecting nonalcoholic beverages. Given that faster reaction times on alcohol trials suggest an attentional bias toward alcohol, a positive bias score thus indicated a bias toward alcohol.

**Table 1:** Visual search task block distribution.

Block	Target picture (1)	Nontarget pictures (15)
1	Active, alcohol related	Active, not alcohol related
2	Passive, alcohol related	Passive, not alcohol related
3 and 4	5-petaled flower	7-petaled flowers
5	Active, not alcohol related	Active, alcohol related
6	Passive, not alcohol related	Passive, alcohol related

**Figure 1:**
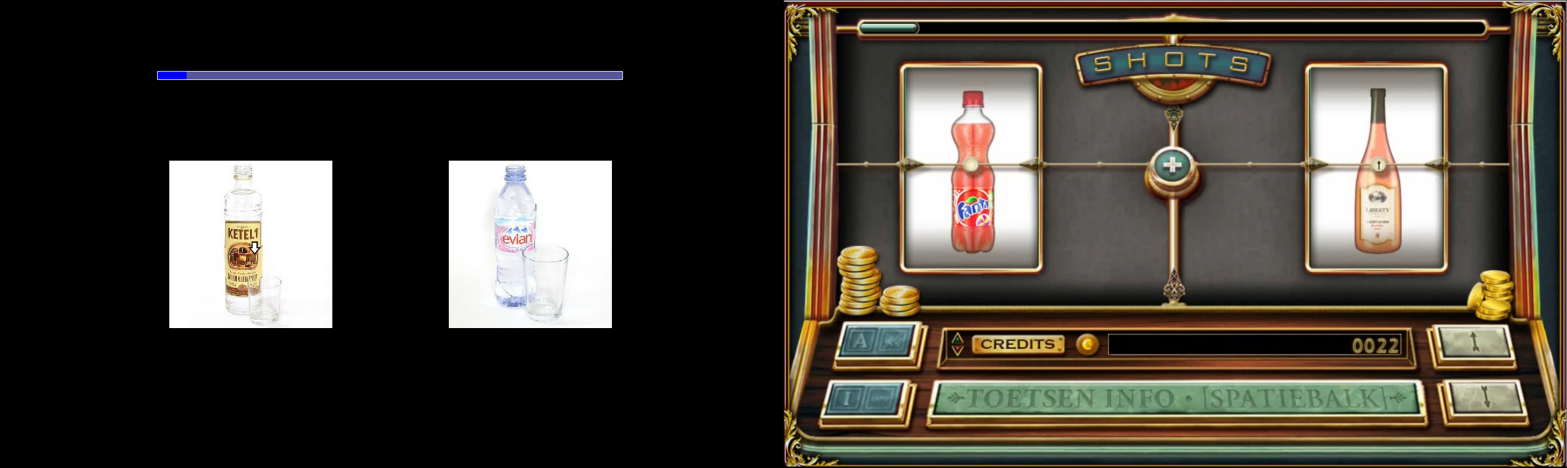
The regular visual probe task (left) and the Shots game implementation (right). The arrow size was matched between the tasks but is enhanced slightly in the left panel (visual probe task) for visibility in print.

In the VPT, participants were shown pairs of alcohol and nonalcoholic beverages followed after 500 milliseconds by a small arrow probe in the location of one of the pictures, pointing upward or downward. The instruction was to press the keyboard’s arrow key corresponding to the arrow’s direction as quickly and accurately as possible. The task consisted of 168 critical trials with pairs of beverages and 32 filler trials with pairs of neutral objects (office supplies), presented in random order over 3 blocks: a starting block of 10 neutral practice trials, then 2 test blocks of 100 trials (84 critical trials). In one of the two test blocks, the pictures disappeared as the arrow became visible (“Go” block); in the other block the pictures remained visible as the arrow probe was superimposed (“Stay” block). The Stay trials were included as they might better detect difficult disengagement from alcohol cues [31,32], while the standard Go trials may be a better measure of rapid allocation and maintenance of attention on alcohol cues. The order of these blocks was counterbalanced over participants. Location of alcohol picture (left or right) and arrow (at alcohol-related or not-alcohol-related stimulus) was fully counterbalanced. The filler trials were included to slightly mask the contingency between arrow placement and the content of the pictures, as well as maintain participants’ attention on the task and avoid anticipatory responses. When an incorrect response was given, the trial had to be redone after feedback was given, and the arrow direction was reapplied randomly. A progress bar indicated the number of trials left in each block. All picture pairs were matched by size and colors (see Figure 1). All stimuli originated from the Amsterdam Beverage Picture Set (ABPS [33]). Attentional bias scores for Stay and Go trials were computed by respectively subtracting the average reaction time for correct trials with the arrow at the location of the alcoholic picture from the average reaction time on correct trials where the arrow was at the location of the nonalcoholic picture. Correction trials following incorrect responses were excluded, as reaction times on those trials were deemed unreliable owing to the stimuli being the same as in the previous trials; reaction times greater than 3000 milliseconds were coded as too late and were also excluded. Given that faster responses on alcohol trials suggest an attentional bias toward alcohol, a positive bias score thus indicated a bias toward alcohol.

### Training

The VPT training tasks were identical to the assessment version, except that after the practice block there was only 1 training block of 156 critical and 16 filler trials, where Stay and Go trials were presented randomly. Additionally, in the VPT-R and VPT-G the arrow always appeared at the location of the nonalcoholic beverage, thus training attention away from the alcoholic stimuli. In the VPT-G condition, participants trained using the Shots game (S van Schie, unpublished data, 2014). The Shots game was functionally identical to the VPT-R training, while looking like a slot machine game with 2 spinning wheels (see Figure 1; note that although the Shots game looks like a slot machine, it has no gambling elements to it. This was also explained to the participants). The game elements used here constitute an *integrated gamification* of the VPT paradigm as defined by the CBM gamification model by Boendermaker and colleagues [22]. It mainly uses a coin-based reward system (see Step 1 [22]) and nicer looking graphics, animations (eg, the spinning wheels with pictures of beverages), and sound effects (eg, when spinning the wheels or pressing a button; see Step 3 [22]). The participant is rewarded for correct and fast responses (using time bonuses and special bonus trials), requiring a coin in order to spin the wheels (ie, start a new trial) and eventually the possibility of reaching a new level (a new look for the machine). The game used picture stimuli similar to those in the ABPS but slightly edited to fit the graphical style of the game.

## Results

### Attrition

After the first training session, 2 participants in the VPT-R condition dropped out of the study and were excluded from the training effect analyses. An additional 6 participants failed to do the follow-up assessment and were excluded from the TLFB training effect analysis. Furthermore, baseline data from 2 participants on the RCQ question 3, and 1 participant on the VPT, were missing because of technical problems and therefore excluded from the relevant analyses.

### Sample Characteristics

At baseline, participants had consumed an average of 15.09 standard units of alcohol (SD 11.46) during the previous 7 days and binged on average on 6.65 occasions (SD 3.48) during the previous 30 days. The AUQ average HAC score was 230.91 (SD 17.17) and with 94% (90/96) of participants scoring ≥8 and 71% (68/96) scoring ≥11, indicating hazardous drinking in a large proportion of the sample [27,28]. See Table 2 for an overview of baseline characteristics. No significant group differences were detected at baseline.

### Training Effects

All dependent variables were screened for univariate extreme outliers based on inspection of stem-and-leaf and box plots. When they were present, or one of the general linear model (GLM) assumptions were violated, a nonparametric method for factorial repeated-measures analysis of variance was used: the Aligned Rank Transform analysis of variance [34], in which data are aligned and then ranked as a preprocessing step, before applying GLM procedures (these results are marked with *).

#### Attentional Bias Change (H1)

There was a significant reduction in alcohol attentional bias over time on the VPT-Go trials, *F*_1,90_*=9.407, *P*=.003, *η*_p_^2^=0.095, as well as a significant interaction with condition, *F*_2,90_*=8.685, *P*<.001, *η*_p_^2^=0.162. Tukey-adjusted contrasts indicated this was due to a significant decrease in bias in the VPT-R condition over time, *t*_90_=3.094, *P*=.031, *r*=.310, and also confirmed the result presented in Table 2 that the VPT-Go bias score at baseline was significantly higher in the VPT-R condition, compared with the VPT-P condition, *t*_179.05_=3.055, *P*=.031, *r*=.223. The VPT-Stay trials also showed a significant reduction in bias over time, *F*_1,90_*=10.894, *P*=.001, *η*_p_^2^=0.108, without an interaction with condition. In contrast, no significant changes over time were found on the VST. A significant overall difference was found between the conditions on the VST-ACT trials: *F*_2,91_=5.480, *P*=.006, *η*_p_^2^=0.107; but post hoc analyses revealed no significant contrasts.

#### Behavioral Change (H2)

There was no significant reduction in TLFB scores for both binges and total use (*P*>.05).

**Table 2:** Baseline characteristics by group.

Characteristics	Placebo (VPT^a^-P)	Regular (VPT-R)	Game (VPT-G)	Total	*P*
Total participants (male)	33 (10)	30 (8)	33 (10)	96 (28)	.936
Age in years, mean (SD)	21.4 (2.1)	21.3 (1.4)	21.0 (2.0)	21.2 (1.8)	.743
AUDIT^b^ score, mean (SD)	13.7 (5.5)	13.5 (3.9)	13.6 (4.9)	13.6 (4.8)	.986
TLFB^c^ (drinks/7 days), mean (SD)	15.7 (14.2)	13.4 (7.6)	16.0 (11.4)	15.1 (11.5)	.634
TLFB (binges/30 days), mean (SD)	6.9 (4.3)	6.3 (2.8)	6.7 (3.3)	6.7 (3.5)	.809
AUQ^d^ (HAC^e^), mean (SD)	229.7 (17.8)	233.9 (20.5)	229.2 (12.4)	230.9 (17.2)	.516
RCQ^f^ -1, mean (SD)	2.2 (0.8)	2.3 (0.7)	2.5 (0.9)	2.4 (0.8)	.454
RCQ-2, mean (SD)	3.8 (1.9)	4.0 (1.9)	3.7 (1.9)	3.8 (1.9)	.682
RCQ-3, mean (SD)	1.8 (0.7)	2.0 (0.6)	1.9 (0.7)	1.9 (0.7)	.499
MTQ^g^, mean (SD)	15.8 (2.4)	16.7 (1.8)	16.4 (2.0)	16.3 (2.1)	.249
VPT-Go^h^ alcohol bias (ms^i^), mean (SD)	−3.7 (20.8)	10.2 (25.1)	0.5 (22.9)	2.1 (23.4)	.053
VPT-Stay^j^ alcohol bias (ms), mean (SD)	−0.2 (37.2)	1.9 (26.1)	3.2 (36.0)	1.6 (33.3)	.920
VST-ACT^k^ alcohol bias (ms), mean (SD)	64.8 (511.8)	250.9 (596.4)	18.9 (495.8)	107.2 (538.0)	.200
VST-PAS^l^ alcohol bias (ms), mean (SD)	116.8 (515.2)	118.3 (536.1)	232.7 (594.4)	157.1 (547.0)	.624

^a^VPT: visual probe task.

^b^AUDIT: Alcohol Use Disorders Identification Test.

^c^TLFB: Timeline Followback.

^d^AUQ: Alcohol Use Questionnaire.

^e^HAC: habitual alcohol consumption.

^f^RCQ: Readiness to Change Questionnaire (items 1, 2, and 3).

^g^MTQ: Motivation to Train Questionnaire.

^h^VPT-Go: VPT trials where the stimulus picture disappeared when the probe appeared.

^i^ms: milliseconds.

^j^VPT-Stay: VPT trials where the stimulus picture remained visible when the probe appeared.

^k^VST-ACT: visual search task with active beverage-related stimuli.

^l^VST-PAS: visual search task with passive beverage-related stimuli.

#### Motivation to Train (H3)

The MTQ demonstrated sufficient internal consistency, Cronbach alpha=.69. Exploratory principal axis factor analysis indicated a single factor. Therefore, the sum score was analyzed. There was a significant decrease in motivation to train over time, *F*_1,91_*=54.377, *P*<.001, *η*_p_^2^=0.374, with no interaction with condition, indicating that motivation decreased similarly in all conditions. Participants’ responses on the EVAL questions only differed between conditions on the question whether they would like to do more training sessions (EVAL-4), *H*_2_=9.987, *P*=.007. Contrasts indicated that the VPT-G conditions scored significantly lower than both the VPT-P condition, *U*=356.0, *P*=.011, *r*=−.313, and the VPT-R condition, *U*=273.0, *P*=.004, *r*=−0.364.

#### Motivation to Change (H4)

The RCQ showed an overall increase in the degree to which participants planned to drink less after the training (a *lower* score on RCQ-2), *F*_1,91_*=5.863, *P*=.017, *η*_p_^2^=0.061. However, there also was a significant interaction between time and condition, *F*_2,91_*=3.865, *P*=.024, *η*_p_^2^=0.078. Tukey-adjusted contrasts indicated a lower motivation to drink less over time for the VPT-G condition, *t*_91_=−2.985, *P*=.041, *r*=.299. The other RCQ items did not show significant effects. See Table 3 for an overview of estimated marginal and interaction means.

#### Additional Training Analyses

Participants differed in terms of the number of errors made during all training sessions, *H* (2)=9.093, *P*=.011, with participants in the VPT-G condition making more errors (mean 40.48, SD 25.91) than those in the VPT-P condition (mean 23.00, SD 11.96), *U*=781.0, *P*=.002, *r*=.374. The average reaction times over all sessions also differed significantly between all training conditions, *H* (2)=59.421, *P*<.001, with the VPT-P condition (mean 557.75, SD 33.05) being slower than the other conditions and the VPT-G condition (mean 373.71, SD 78.18) being faster than the other conditions (VPT-R, mean 516.49, SD 50.52).

**Table 3:** Training effects—estimated means.

Training effect		Placebo (VPT^a^-P)	Regular (VPT-R)	Game (VPT-G)	Time
**TLFB^b^(drinks/7 days), mean (SE)**					
	Baseline	124.4 (14.4)	157.5 (14.5)	137.7 (13.7)	134.3 (8.2)
	Posttraining	130.7 (14.4)	129.2 (14.5)	120.3 (13.7)	133.2 (8.2)
	2-Week follow-up	127.9 (14.4)	127.1 (14.5)	137.8 (13.7)	130.0 (8.2)
	Condition	133.9 (11.5)	125.0 (11.6)	138.7 (11.2)	
**TLFB (binges/30 days), mean (SE)**					
	Baseline	128.9 (14.4)	139.3 (14.6)	140.5 (13.9)	136.4 (8.2)
	Posttraining	122.2 (14.4)	138.2 (14.6)	133.4 (13.9)	135.6 (8.2)
	2-Week follow-up	129.1 (14.4)	121.3 (14.6)	139.5 (13.9)	125.5 (8.2)
	Condition	134.5 (12.7)	122.9 (12.7)	140.1 (12.3)	
**MTQ^c^, mean (SE)**					
	Baseline	92.9 (9.7)	94.8 (10.1)	100.7 (9.7)	113.6 (5.3)
	Posttraining	96.0 (9.7)	90.7 (10.1)	91.9 (9.7)	75.4 (5.3)
	Condition	85.0 (8.5)	102.9 (8.75)	95.6 (8.5)	
**RCQ^d^ -1, mean (SE)**					
	Baseline	106.3 (9.5)	76.0 (9.9)	91.6 (9.5)	99.0 (5.5)
	Posttraining	110.4 (9.5)	89.3 (9.9)	93.5 (9.5)	90.0 (5.5)
	Condition	86.5 (8.4)	99.9 (8.7)	97.2 (8.4)	
**RCQ-2, mean (SE)**					
	Baseline	108.2 (9.5)	76.1 (9.8)	86.5 (9.5)	100.4 (5.6)
	Posttraining	100.8 (9.5)	85.4 (9.8)	110.1 (9.5)	88.6 (5.6)
	Condition	84.3 (8.4)	101.4 (8.7)	97.7 (8.4)	
**RCQ-3, mean (SE)**					
	Baseline	91.6 (9.2)	69.8 (9.6)	88.6 (9.3)	97.5 (5.4)
	Posttraining	116.9 (9.2)	83.4 (9.6)	104.7 (9.3)	87.5 (5.4)
	Condition	84.1 (7.4)	103.1 (7.6)	90.2 (7.5)	
**VPT-Go^e^ alcohol bias (ms^f^), mean (SE)**					
	Baseline	77.1 (9.2)	118.1 (9.7)	80.9 (9.3)	104.8 (5.5)
	Posttraining	105.0 (9.2)	76.5 (9.7)	103.3 (9.3)	82.2 (5.5)
	Condition	91.7 (7.0)	87.8 (7.2)	101.0 (7.0)	
**VPT-Stay^g^ alcohol bias (ms), mean (SE)**					
	Baseline	79.8 (9.5)	103.3 (10.0)	92.6 (9.6)	106.9 (5.5)
	Posttraining	105.6 (9.5)	88.6 (10.0)	91.0 (9.6)	80.1 (5.5)
	Condition	99.6 (6.5)	88.4 (6.7)	92.4 (6.6)	
**VST-ACT^h^ alcohol bias (ms), mean (SE)^i^**					
	Baseline	64.8 (93.5)	226.6 (101.5)	18.9 (93.5)	103.4 (55.6)
	Posttraining	50.2 (104.7)	535.1 (113.7)	28.0 (104.7)	204.4 (62.2)
	Condition	57.5 (79.7)	380.8 (86.5)	23.5 (79.7)	
**VST-PAS^j^ alcohol bias (ms), mean (SE)^i^**					
	Baseline	116.8 (95.8)	153.6 (104.0)	232.7 (95.8)	167.7 (56.9)
	Posttraining	29.6 (96.8)	178.9 (105.1)	235.5 (96.8)	147.9 (57.5)
	Condition	73.2 (75.7)	166.1 (82.2)	234.1 (75.7)	

^a^VPT: visual probe task.

^b^TLFB: Timeline Followback (shows the number of standardized drinks during the week before the pretraining assessment).

^c^MTQ: Motivation to Train Questionnaire.

^d^RCQ: Readiness to Change Questionnaire (questions 1, 2, and 3).

^e^VPT-Go: VPT trials where the stimulus picture disappeared when the probe appeared.

^f^ms: milliseconds.

^g^VPT-Stay: VPT trials where the stimulus picture remained visible when the probe appeared.

^h^VST-ACT: visual search task with active beverage-related stimuli.

^i^Using regular analysis of variance procedure. For the all the other values, the Aligned Rank Transform procedure was used.

^j^VST-PAS: visual search task with passive beverage-related stimuli.

## Discussion

### Principal Findings

This study aimed to decrease attentional bias toward alcohol and hazardous drinking behavior in young adults by using a CBM-A training with game elements. After training, there was an overall decline in attentional bias on the VPT task, but this effect was primarily driven by the regular VPT training condition, where a stronger bias was observed at baseline. The training effect did not generalize to the VST task, nor was there a decline in alcohol use after the training. Motivation to train decreased equally in all conditions, indicating that the training indeed became boring over time, but also that the motivational elements of the Shots game could not sufficiently counteract this effect. Interestingly, motivation to change, with respect to planning to drink less in the future, increased in the regular and placebo training but decreased in the game training condition. Moreover, participants in the game condition indicated a lower motivation to continue training compared with the other conditions. Participants also differed with regard to overall speed and accuracy of responses during the training, which may be due to the more complex nature of the gamified version of the training or the level of engagement in the training.

These findings regarding the motivational effects of the training may have important implications on the potential risks involved with using certain types of game elements. Although the gamified training arguably looked fancier than the regular training, the game elements in this study merely consisted of upgraded visuals and a coin-based reward system. There was no story line and only limited progression and personalization options were available in the game. As such, it is likely that these rather minimal game elements alone were insufficient to increase motivation to train in our student sample. Moreover, most participants will likely compare any gamified training with what they believe a game experience should look and feel like [22]. If the game training experience then disappoints, motivation could indeed take a dive, even going below the level observed in the regular training conditions, because expectations were higher to begin with. This was perhaps reflected in our finding that participants in the game condition, specifically, were less motivated to continue training, as well as start drinking less, after the training. Finally, these results may also be related to the visual and auditory game elements used, which might have distracted participants from the training elements, rendering it less effective. Indeed, the standard training condition with no game elements did show a small change in attentional bias. Moreover, motivation to change increased only in the nongame conditions. It could be that the exposure to alcohol cues gave participants a push toward a readiness to change but only when there were no distracting game elements surrounding those cues. Although this last point is speculative, it is clear that some game elements may be detrimental not only to motivation to train but also to the training mechanisms themselves. More research is necessary in order to determine which game elements are best suited for cognitive training, and CBM-A in particular. For example, more levels, a background story, or the introduction of character development throughout the multiple sessions of the game could benefit participants’ motivation. However, such additional elements would also have to be tested to see what their effect is on cognitive bias and related behavior.

### Limitations

Some limitations apply to this study. First, despite hazardous drinking in a substantial part of the sample, the modest training effects in this sample may be partially due to a relatively small alcohol attentional bias at baseline, which is a known moderator of training effects [13,35,36]. However, it should be noted that this notion does not necessarily make CBM-A inappropriate in such samples. For example, Schoenmakers and colleagues [21] detected no bias at baseline in their sample but still found a positive bias after training in their control group and a negative bias in their experimental group. Furthermore, this study included a total of 624 critical training trials divided over 4 sessions. Although this number is similar to that used in other research (eg, [19]), other attentional bias modification studies have used markedly larger numbers (eg, Schoenmakers et al [21], where participants completed 2640 training trials over 5 sessions). Given the very likely dose-response relationship between use and effectiveness of cognitive training paradigms, the amount of training practice may have prevented the training from efficiently changing attentional bias. Finally, a recent meta-analysis [37] concluded that Web-based CBM-A studies usually show smaller effect sizes than laboratory-based studies. Although the assessments took place in the laboratory, it is possible that the option to train at home had a negative effect on participants’ motivation, for example, by making participants take the training less seriously.

### Conclusions

In sum, the novel game-like approach used in this study proved insufficient to motivate young adults to train, in comparison with a regular CBM-A training. In fact, some aspects of motivation appeared to deteriorate rather than improve, suggesting that gamification can have drawbacks if not done optimally. It could be concluded from this study that a point-based reward system in combination with fancy graphics does not satisfy participants’ expectations of what constitutes a game. Because one expects a game to be fun, this may have detrimental effects on motivation. Moreover, when those game elements distract participants from the training elements, they may actually impair performance. A second notion that can be taken from this study is that the observed attentional biases toward alcohol as measured with the VPT in this heavy drinking student sample were remarkably low. Whether this has implications for the presence of attentional bias in adolescent samples in general or merely pertains to the VPT paradigm as a valid assessment measure of attentional bias remains to be determined by future research. If nothing else, however, these results underscore the importance of careful scientific evaluation before serious games are used as interventions.

## Multimedia Appendix 1

Motivational questions.

## Abbreviations

ABPS: Amsterdam Beverage Picture Set
ADHD: attention-deficit/hyperactivity disorder
AUDIT: Alcohol Use Disorders Identification Test
AUQ: Alcohol Use Questionnaire
CBM-A: cognitive bias modification of attention
GLM: general linear model
HAC: habitual alcohol consumption
MTQ: Motivation to Train Questionnaire
RCQ: Readiness to Change Questionnaire
TLFB: Timeline Followback
VPT: visual probe task
VPT-G: gamified VPT training
VPT-P: placebo VPT training
VPT-R: regular VPT training
VST: visual search task
