# Supplementary material for: Attentional Bias Modification With Serious Game Elements: Evaluating the Shots Game
Source: JMIR Serious Games. 2016 Dec 6;4(2):e20. doi: 10.2196/games.6464 (PMC5174726; doi:10.2196/games.6464)
Supplement: Supplementary file 1 [file games_v4i2e20_app1.pdf]

## Multimedia Appendix 1 – Motivational Questions

| Item | Dutch (original) | English (translation) |
|---|---|---|
| MTQ 1 | Ik ben gemotiveerd om aan deze training mee te gaan doen. | I am motivated to participate in this training. |
| MTQ 2 | Ik geloof dat deze training mij kan helpen om meer controle te krijgen over mijn drinkgedrag. | I believe that this training can help me to get more control over my drinking behavior. |
| MTQ 3 | Ik wil graag mijn best doen op de training. | I want to do my best on the training. |
| MTQ 4 | Ik vind de training zonde van mijn tijd. | I think the training is a waste of my time. |
| RCQSA 1 | Denk je dat je meer drinkt dan goed voor je is? | Do you think that you drink more than is good for you? |
| RCQSA 2 | Ben je van plan minder te gaan drinken? | Are you planning to drink less? |
| RCQSA 3 | Ben je in de afgelopen drie maanden veranderd wat betreft je drinken? | Did you change your drinking behavior during the past 3 months? |
| EVAL 1 | Ik denk dat deze training mensen in het algemeen kan helpen om meer controle te krijgen over hun drankgebruik. | I think this training can help people in general to get more control over their alcohol use. |
| EVAL 2 | Ik vond het leuk om deel te nemen aan dit onderzoek. | I liked participating in this study. |
| EVAL 3 | Ik zou deze training aanraden aan een vriend/vriendin met een soortgelijk drinkgedrag. | I would recommend this training to a friend who has a similar drinking behavior. |
| EVAL 4 | Ik zou graag nog meer trainingssessies willen doen. | I would like to do more training sessions. |
| EVAL 5 | Ik vond 4 trainingsessies te veel. | I thought 4 training sessions were too many. |
| EVAL 6 | De vormgeving van de training motiveerde me om echt mijn best te doen. | The environment of the training motivated me to really do my best. |
